# Process Intensification of Unripe Plantain Peel UV-C-Assisted Hot-Air Drying Combined with Ultrasound and Oxalic Acid Pretreatments: Drying Kinetics, Microstructure, and Product Quality

**DOI:** 10.3390/foods15173140

**Published:** 2026-09-04

**Authors:** Adriano S. H. de Souza, Eduarda M. de Souza, Fernanda G. da Silva, Ana M. R. B. da Silva, João H. F. da Silva, Patrícia M. Azoubel

**Affiliations:** 1 Departamento de Engenharia Química, Universidade Federal de Pernambuco, Av. Prof. Arthur de Sá, s/n, Cidade Universitária, Recife 50740-521, PE, Brazil; adriano.honoratosouza@ufpe.br (A.S.H.d.S.); eduarda.mariasouza@ufpe.br (E.M.d.S.); ana.rbsilva@ufpe.br (A.M.R.B.d.S.); 2 Departamento de Nutrição, Universidade Federal de Pernambuco, Av. Moraes Rego, s/n, Cidade Universitária, Recife 50670-901, PE, Brazil; fernanda.goncalvessilva@ufpe.br; 3 Engenharia de Alimentos, Universidade Federal do Agreste de Pernambuco, Av. Bom Pastor, s/n, Cidade Universitária, Garanhuns 55292-270, PE, Brazil; silva.jhf@ufape.edu.br

**Keywords:** process intensification, plantain peel, mass transfer, moisture diffusivity, bioactive preservation

## Abstract

The agro-industrial valorization of unripe plantain peels through flour production represents a sustainable strategy for waste reduction and nutrient recovery. This study investigated the process intensification of plantain peel drying by evaluating the combined effects of UV-C-assisted hot-air drying with ultrasound and oxalic acid pretreatments. A 2^3^ full factorial design was employed to evaluate the effects of UV-C lamp distance, ultrasound time and oxalic acid concentration on drying kinetics, effective moisture diffusivity, and the retention of bioactive compounds. The combination of the most intense levels of the pretreatments with a 9 cm distance between the radiation source and the sample achieved a 40.68% reduction in drying time compared to the control (without pretreatments). Among the mathematical models tested, the Logarithmic model provided the most accurate fit (R^2^ > 0.99), effectively describing the falling-rate period and mass transfer phenomena. Scanning electron microscopy revealed structural modifications, including microchannels and surface pores, consistent with enhanced moisture transport and increased effective moisture diffusivity. The accelerated drying kinetics also led to higher contents of bioactive compounds, including total phenolics, ascorbic acid, and carotenoids, while maintaining adequate water activity and color stability. These findings demonstrate the combined potential of UV-C radiation, ultrasound, and oxalic acid to intensify drying efficiency while improving the functional quality of unripe plantain peel flour.

## 1. Introduction

The global food industry generates substantial quantities of agro-industrial by-products, with fruit processing alone accounting for tonnes of peels, seeds, and pulp residues annually. Among these, plantain (*Musa paradisiaca* L.) peels represent a significant valorization opportunity, as they are rich in dietary fiber, polyphenols, and carotenoids, compounds with demonstrated antioxidant and functional properties [1,2,3,4,5]. However, the conversion of these by-products into shelf-stable, high-value bioproducts remains constrained by conventional drying technologies, which are energy-intensive and often result in substantial losses of thermolabile bioactive compounds [6,7,8].

Convective drying, the predominant industrial method for fruit residue dehydration [1], operates within fundamental mass transfer limitations [9,10]. The process is governed by two sequential resistances: external resistance at the solid–air interface (boundary layer) and internal resistance within the product matrix (diffusion-limited transport) [11,12]. These resistances directly determine the drying kinetics and, consequently, the thermal exposure time, a critical parameter for bioactive preservation [1,13]. Reducing processing time while maintaining or enhancing product quality requires a deliberate engineering strategy that addresses both resistances simultaneously.

Process intensification through the integration of complementary pretreatments and assisted drying technologies offers a promising pathway to overcome these limitations. Ultrasound-assisted pretreatment induces cavitation phenomena that disrupt cellular structures, creating microchannels and increasing porosity [14,15]. This structural modification directly enhances internal moisture diffusivity, reducing the internal resistance to mass transfer [16,17,18,19].

Concurrently, UV-C radiation, when applied during the drying process rather than solely as a pretreatment, acts as a continuous energy source at the product surface, potentially reducing the external resistance and accelerating evaporation rates [20]. Silva et al. [21] reported that UV-C-assisted drying, combined with ultrasound and acetic acid as a pretreatment, reduces the drying time required for unripe bananas to reach equilibrium moisture content. Similarly, Köse and Erentürk [22] observed that UV radiation significantly increased drying rates during convective drying of mistletoe leaves. Nevertheless, the implications of UV-C treatment for retaining bioactive compounds during drying remain insufficiently understood, particularly in banana peel, which contains approximately 32% lignin, 21% cellulose, and 17% hemicellulose [23]. This composition, combined with the lack of studies, highlights plantain peel as a suitable model for examining UV-C-induced transformations during dehydration.

Furthermore, chemical pretreatments with organic acids (such as oxalic acid) further contribute to structural degradation through hydrolysis of starch granules and cell wall components, while simultaneously inhibiting enzymatic browning and stabilizing the antioxidant matrix [24,25]. The literature reports several successful applications of organic acid solutions as pretreatments in drying processes, including ascorbic acid for *Gardenia erubescens* fruits [26]; acetic and citric acids for pumpkin [27]; ascorbic, citric, and acetic acids for banana [28]; and citric acid for potato [29] and bean seeds [30]. Although oxalic acid has been investigated in the context of lignocellulosic biomass pretreatment for biofuel production [31], its application for improving drying processes remains largely unexplored.

Despite the individual efficacy of these technologies, research investigating their combined application within a multi-hurdle approach during the drying of agro-industrial residues remains limited. Specifically, the mechanisms by which UV-C radiation assists mass transfer when combined with structural modifications induced by ultrasound and chemical pretreatment have not been systematically elucidated. Furthermore, the quantitative relationship between process intensification parameters (UV-C distance, ultrasound exposure, acid concentration) and both drying kinetics and bioproduct quality remains poorly characterized in the literature.

This study addresses this knowledge gap by investigating the combined intensification of unripe plantain peel drying through a multi-hurdle approach integrating UV-C-assisted convective drying with ultrasound and oxalic acid pretreatments. A 2^3^ full factorial design was employed to evaluate the influence of three process variables on drying kinetics, effective moisture diffusivity, and bioproduct quality attributes. The research aims to: (1) quantify the individual and interactive effects of UV-C distance, ultrasound exposure time, and oxalic acid concentration on drying time and mass transfer phenomena; (2) develop accurate mathematical models describing the falling-rate period of plantain peel drying; (3) elucidate the structural mechanisms underlying process intensification through scanning electron microscopy; and (4) demonstrate that accelerated drying kinetics, achieved through multi-hurdle intensification, preserve higher levels of bioactive compounds and functional properties. The outcomes of this work provide a robust engineering framework for the sustainable valorization of plantain by-products into functional bioproducts with enhanced nutritional and antioxidant attributes.

## 2. Materials and Methods

### 2.1. Raw Material

Unripe plantain (*Musa paradisiaca*) was purchased from a local market in Jaboatão dos Guararapes, Pernambuco, Brazil. The fruits were manually peeled using a stainless-steel knife, and the peels were separated from the pulp. For process standardization, the peels were cut into uniform pieces (2.0 × 2.0 × 0.5 cm) prior to processing.

### 2.2. Experimental Design

A 2^3^ full factorial design (Table 1) was employed to define the drying conditions. The independent variables were: distance between the UV-C radiation source and the sample (D), ultrasound treatment time (U), and oxalic acid concentration (AO). The response variable was the time required for the samples to reach a moisture content of 15% (wet basis, t_15%_), in accordance with Brazilian regulations for flour products [32]. Because the 2^3^ factorial design allows the screening of main and interaction effects but does not estimate quadratic (curvature) terms, the conditions identified in this study should be regarded as promising combinations to be confirmed by response-surface optimization and experimental validation in future work.

### 2.3. Ultrasound and Oxalic Acid Pretreatments

According to the experimental design, when ultrasound pretreatment was applied, plantain peel samples were immersed in a 250 mL beaker containing distilled water at a sample-to-water ratio of 1:4 (*w*/*w*) [33]. The beaker was then placed in an ultrasonic bath (Ultronique, Q 9.5/37, Indaiatuba, Brazil) operating at 37 kHz, without mechanical agitation [34], for the times specified in Table 1. After treatment, samples were drained and gently dried with absorbent paper for 10 s.

For oxalic acid (Neon, Suzano, Brazil) pretreatment, samples were immersed in a 250 mL beaker containing aqueous oxalic acid solutions at the concentrations defined in Table 1, maintaining the same 1:4 (*w*/*w*) ratio. The immersion time was 10 min. After immersion, the samples were taken out from the solution, rinsed with distilled water to remove the residual acid from the surface, followed by surface drying with absorbent paper for 10 s [35].

When both treatments were applied, ultrasound pretreatment was performed first, immediately followed by immersion in the oxalic acid solution. The residual oxalate content of the flour was not measured in this study.

### 2.4. Drying

Drying experiments were conducted in a horizontal fixed-bed dryer using air at 60 °C and a velocity of 4.3 m/s. Two UV-C lamps (OSRAM HNS L 36W 2G11) emitting radiation in the 200–280 nm range (peak at 254 nm) were positioned at distances defined in Table 1. A quartz window installed at the top of the drying chamber allowed UV radiation to reach the samples. More details of the drying equipment were described by Silva et al. [21].

The irradiance at the sample surface was not directly measured, since a calibrated UV-C radiometer was not available in our laboratory, and no estimates of the irradiance or of the cumulative UV-C fluence were made. The UV-C distance (D) was therefore used as the process variable, as defined in the experimental design.

Sample mass was recorded every 1 min using a semi-analytical balance (Shimadzu^®^, UW4200H, Kyoto, Japan) coupled to the drying tray, with data acquisition in real time. Drying was continued until constant weight was achieved. Drying kinetics were evaluated by fitting four empirical models (Table 2) to the experimental data using the dimensionless moisture ratio (MR), calculated using Equation (1), with Statistica^®^ version 10.0 software.

(1) $$MR=\frac{X_{t}-X_{e}}{X_{0}-X_{e}}$$

where *MR* is the dimensionless moisture ratio, *X_t_* is the moisture content at time *t* (kg water/kg dry matter), *X_e_* is the equilibrium moisture content (kg water/kg dry matter), *X*_0_ is the initial moisture content (kg water/kg dry matter).

To assess the goodness of fit between the models and the experimental data, the statistical parameters residual sum of squares (*RSS*), root mean square error (*RMSE*), and residual standard deviation (*RSD*) were calculated according to Equations (2)–(4), respectively. The coefficient of determination (R^2^) was also considered.

(2) $$RSS=\sum_{i=1}^{N}{{(M}_{0}-M_{P})}^{2}$$

(3) $$RMSE=\frac{RSS}{ʋ}$$

(4) $$RSD=\sqrt{RMS}$$

where *M*_0_ is the experimental value, *M_P_* is the model-predicted value, *N* is the number of experimental observations, and *ʋ* represents the degrees of freedom of the regression model.

Based on the statistical indicators described above, a mathematical model is considered predictive when the values of *RSS*, *RMSE*, and *RSD* approach zero, while the coefficient of determination (R^2^) is close to unity [36].

To solve Fick’s model as presented by Crank [37], the methodology proposed by Silva et al. [21] was applied, which suggests two approaches for estimating the effective diffusivity (*D_ef_*). In the first approach, *D_ef_* values were estimated at different drying times (1, 5, 10, 15, 25, 50, 75, 100, 125, 150, 180, and 200 min) using the “Goal Seek” function in Microsoft Excel, considering 1000 terms in the series. This procedure aimed to identify a range in which *D_ef_* becomes sufficiently small to be assumed constant, thus characterizing the falling-rate period of the drying process. In this method, diffusivity was defined as the average value (*D_efM_*) over the selected time interval. In the second approach, Equation (5) was used as a thin-layer model truncated at the fifth term of the series. The purpose of using the second approach is to obtain effective diffusivity (*D_ef_*) for the entire period of the falling rate, interpreting the Fick model truncated at the fifth term as an empirical model statistically adjustable to the data. This comparative analysis between *D_ef_* and *D_efM_*, using the same data range, highlights the magnitude of the error between the methods. Experimental data within the previously identified falling-rate period were fitted using Statistica^®^ software (version 10.0, Tulsa, OK, USA).

(5) $$MR=\frac{8}{\pi^{2}}\sum_{i=0}^{\infty }\frac{1}{{\left(2i+1\right)}^{2}}\exp\left[{\left(-2i+1\right)}^{2}\pi^{2}D_{ef}\frac{t}{4l^{2}}\right]$$

where *t* is the time (s), *i* is the number of terms in the series; *D_ef_* is the effective diffusivity (m^2^/s), and *l* is the half-thickness of the sample.

### 2.5. Scanning Electron Microscopy (SEM)

Morphological analysis was performed using scanning electron microscopy (SEM) (Thermo Scientific™ Phenom XL, Eindhoven, The Netherlands) at an accelerating voltage of 15 kV and magnification of 450×. As samples were already dried, no additional preparation was required. The microstructural assessment was qualitative, since quantitative image analysis of pore size and pore density was not performed in this study.

### 2.6. Quality Analyses

Prior to quality analyses, samples were dried until reaching 15% moisture content (wet basis) and then ground using an electric grinder (Kodi, Changzhou, China) to obtain flour. Analyses were performed on both fresh peels and selected flour samples obtained under conditions selected by the factorial design. A control sample dried by hot air at 60 °C without UV-C radiation and without pretreatments was also evaluated to assess the retention of these compounds.

Water activity was measured using a calibrated portable analyzer (Decagon, Pawkit, Pullman, WA, USA) at 25 ± 1 °C. Color parameters (L*, a*, b*) were determined using a portable calibrated colorimeter (Konica Minolta, CM-600D, Sakai, Japan), and total color difference (ΔE) was calculated as:

(6) $$∆E=\sqrt{{\left(L-L_{0}\right)}^{2}+{\left(a^{*}-a_{0}^{*}\right)}^{2}+{\left(b^{*}-b_{0}^{*}\right)}^{2}}$$

where L* and *L*_0_* represent the lightness of the pretreated dried and control samples, respectively, a* and *a*_0_* correspond to the red color intensity of the pretreated dried and control samples, respectively, and b* and *b*_0_* denote the yellow color intensity of the pretreated dried and control samples, respectively.

Bioactive compounds were evaluated by determining ascorbic acid, total carotenoids, total phenolic compounds, and antioxidant capacity. Total phenolic content was determined using the Folin–Ciocalteu (Sigma-Aldrich, Saint Louis, MO, USA) method [38] and expressed as mg gallic acid (Dinamica, Indaiatuba, Brazil) equivalents (GAE)/g dry matter. Ascorbic acid content was quantified according to AOAC [39], based on the reduction of 2,6-dichlorophenolindophenol (Merck, Darmstadt, Germany), and expressed as mg/100 g dry matter. Total carotenoids were determined following Rodriguez-Amaya [40], based on extraction with acetone (Química Moderna, Santana do Parnaíbla, Brazil) and petroleum ether (Anidrol, Diadema, Brazil), and expressed as µg/g dry matter. Antioxidant capacity was evaluated using the DPPH (Sigma-Aldrich, Darmstadt, Germany) radical scavenging method [41], with results expressed as mg Trolox (Sigma-Aldrich, Darmstadt, Germany) equivalents (TE)/g dry matter.

### 2.7. Statistical Analyses

All analyses were performed in triplicate, and results are presented as mean ± standard deviation. Analysis of variance (ANOVA) and Tukey’s test were conducted using Statistica^®^ version 10.0 at a 95% confidence level (*p* < 0.05). Because the factorial runs at the corner points were not replicated, the pure error used to test the significance of the main and interaction effects in the Pareto analysis was estimated from the variance of the three replicates of the center point (11D10U5AO), which provided 2 degrees of freedom for the error term.

## 3. Results and Discussion

### 3.1. Factorial Design Analysis and Drying Time

The intensification of unripe plantain peel drying was systematically evaluated through a 2^3^ full factorial design. The time required for the samples to reach a moisture content of 15% (wet basis), denoted as *t_15_%*, was used as the response variable (Table 3). This parameter was determined from the drying kinetics obtained under the different experimental conditions.

**Figure 1:**
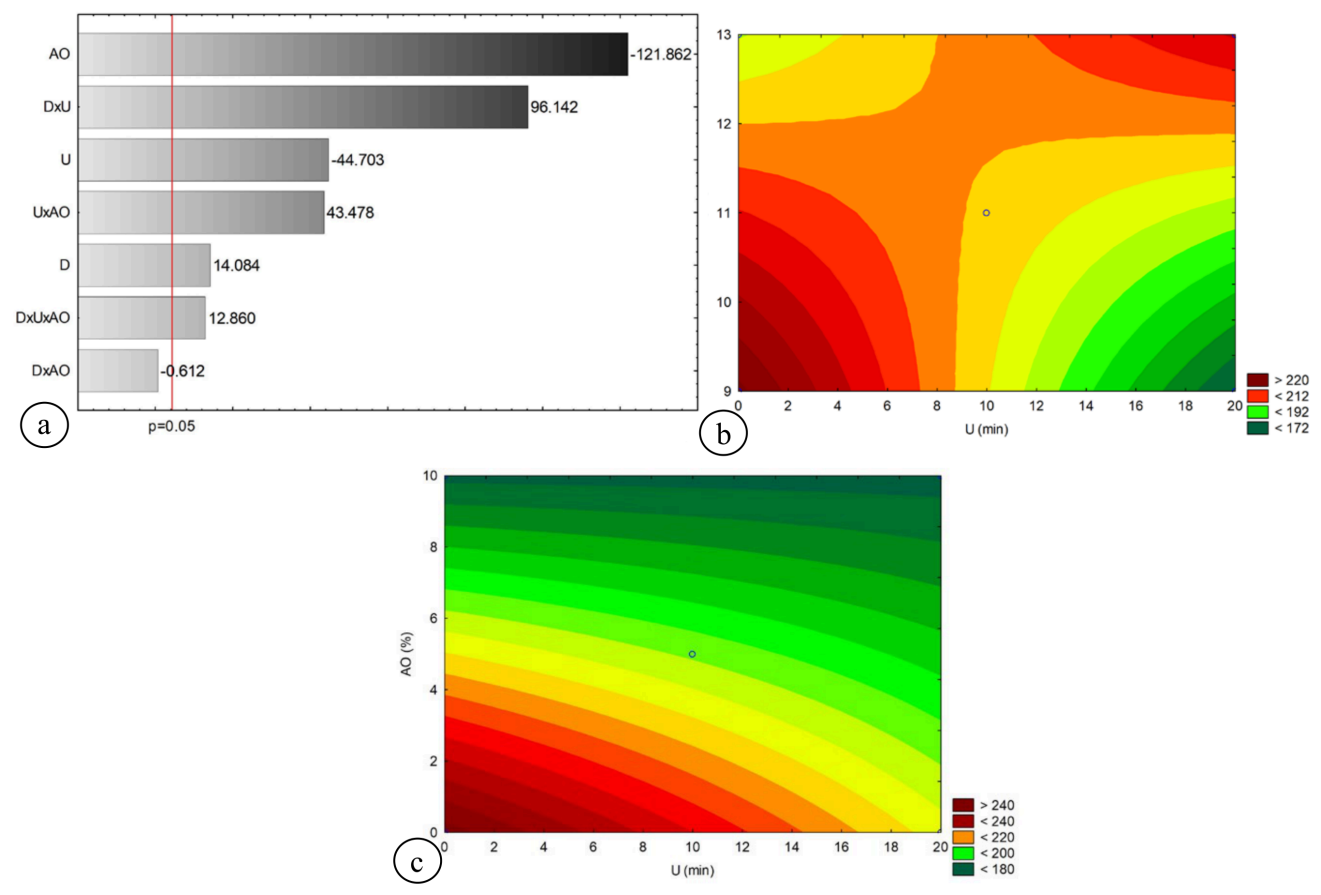
Pareto chart of standardized effects for t_15%_ and surface plots of the combined effects. (**a**) Pareto chart of the standardized effects D (radiation distance), U (ultrasound time), and AO (acid concentration) for t_15%._ (**b**) Surface graphs of the combined D and U effects. (**c**) Surface graphs of the combined U and AO effects.

The drying behavior of unripe plantain peels was significantly affected by the combined application of UV-C-assisted drying and ultrasound-assisted oxalic acid pretreatments. The Pareto chart (Figure 1a) illustrates the statistical significance of the main and interaction effects, while the contour plots (Figure 1b,c) depict the interactions among the evaluated factors. As shown in Figure 1a, all three primary factors—UV-C lamp distance (D), ultrasound exposure time (U), and oxalic acid concentration (AO)—were statistically significant (*p* < 0.05) in reducing the drying time to reach the target moisture content of 15% (wet basis). Among these, oxalic acid concentration emerged as the most influential factor, followed by ultrasound duration and UV-C distance. The negative effects associated with AO and U indicate that increasing these factors individually reduces the drying time required to reach the target moisture content.

The individual application of ultrasound for 20 min (9D20U0AO) and oxalic acid at 10% (9D0U10AO) resulted in drying time reductions of 26.62% and 23.57%, respectively, compared with the control condition (9D0U0AO). Similar intensification trends using ultrasound and organic acids or ethanol have been reported for various biomass matrices [17,18,26,27].

Regarding UV-C radiation distance, the positive effect observed for D (Figure 1a) indicates that shorter distances between the UV-C source and the sample promote faster drying. According to Babu et al. [20], ultraviolet radiation induces photo-oxidative modifications in lignocellulosic materials, altering surface functional groups and generating free radicals that contribute to structural degradation. These changes facilitate moisture migration and evaporation, thereby improving drying efficiency. It should be noted, however, that the irradiance at the sample surface was not directly measured, since a calibrated UV-C radiometer was not available in our laboratory, and no estimates of the irradiance or of the cumulative UV-C fluence were made. Because the drying time differed among treatments, the total UV-C exposure received by each sample is unknown and likely differed among conditions. This limits the interpretation of the UV-C distance effect: the differences in drying time observed between the conditions at 9 and 13 cm may reflect not only the radiation intensity at the sample surface, but also the different total exposure times. The effect of UV-C distance should therefore be regarded as qualitative, and direct radiometric measurement of the surface irradiance and of the cumulative fluence is recommended in future studies.

Although studies evaluating UV radiation during drying remain scarce, several investigations have reported beneficial effects when UV is applied as a pretreatment. Forouzanfar et al. [42] observed reduced drying times in mushrooms exposed to UV-B radiation, attributing this behavior to increased cell wall porosity. Similarly, Phimphilai et al. [43] reported accelerated drying of longan fruits following UV-C pretreatment due to enhanced surface degradation and water evaporation.

The Pareto chart also reveals significant interaction effects for D×U, U×AO, and D×U×AO, whereas the D×AO interaction was not significant. As illustrated in Figure 1b, drying time decreases when ultrasound time is increased while the UV-C distance is reduced, demonstrating a favorable interaction between these factors. Figure 1c shows relatively short drying times at high levels of oxalic acid. The significant interaction terms indicate that the effect of each factor depended on the levels of the other factors; nevertheless, a significant interaction alone does not demonstrate synergy, because the response of the combined treatment was not compared with the additive expectation of the individual effects. The term “combined effect” is therefore used throughout this work. In this context, the condition 9D20U10AO (9 cm UV-C distance, 20 min ultrasound, and 10% oxalic acid) achieved the largest reduction in total drying time (156 min vs. 263 min for the control), demonstrating the efficiency of the multi-hurdle approach.

### 3.2. Drying Kinetics and Mathematical Modeling

**Figure 2:**
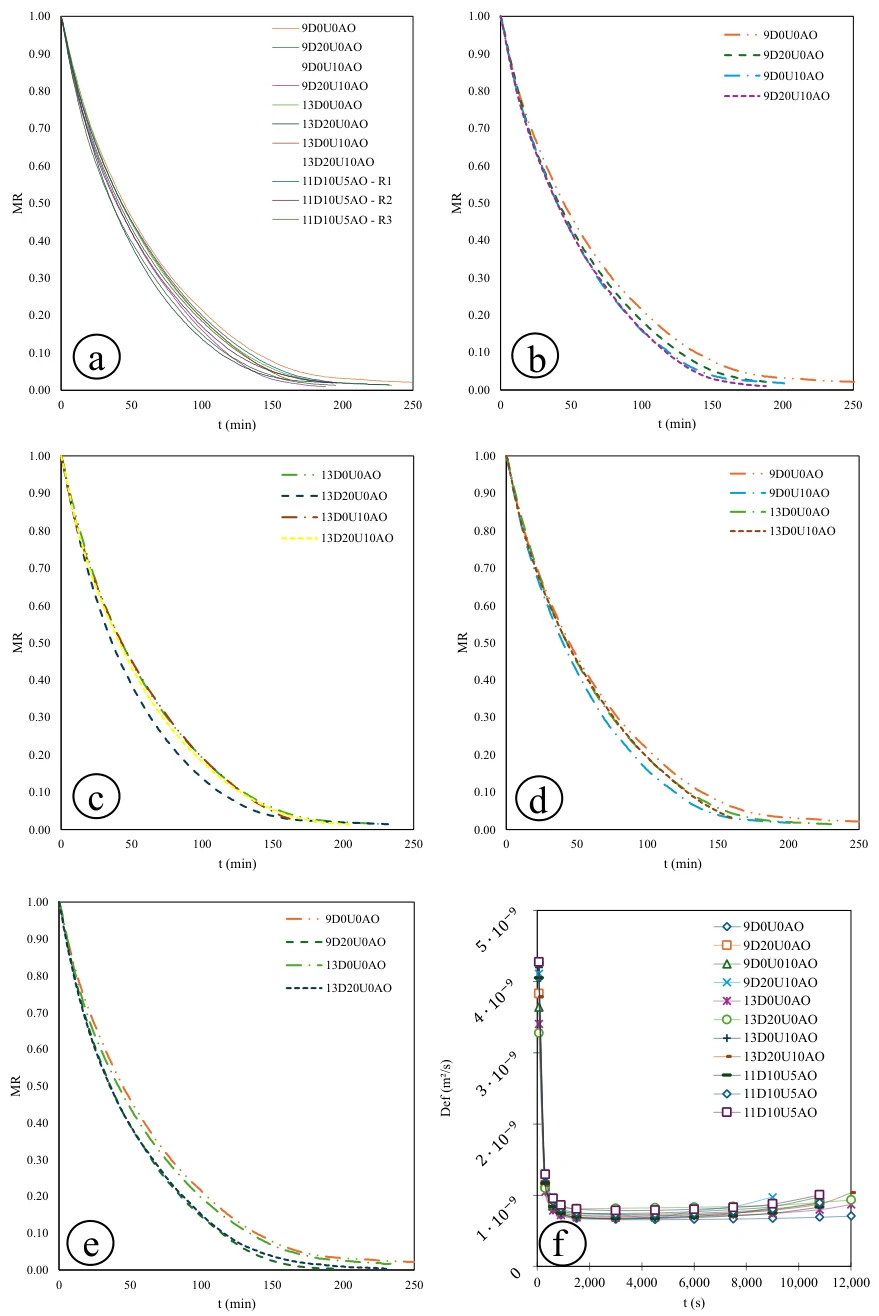
Drying kinetics and behavior of the effective diffusion coefficient (D_ef_) during drying. (**a**) Drying kinetics for all conditions analyzed. (**b**) Drying kinetics at D = 9 cm. (**c**) Drying kinetics at D = 13 cm. (**d**) Drying kinetics without ultrasound. (**e**) Drying kinetics without oxalic acid. (**f**) Effective diffusivity coefficient under all conditions analyzed.

The drying kinetics obtained from the different treatments applied to unripe plantain peels are presented in Figure 2, showing the evolution of the moisture ratio (*MR*) over time. Figure 2a displays all experimental runs defined by the proposed factorial design, including the three replicates at the central point. The observed behavior is typical of drying processes in fruits and vegetables, characterized by a rapid exponential decline at the beginning, followed by a gradual approach to equilibrium near zero as steady-state conditions are reached. This trend indicates that the process is predominantly governed by the falling-rate period [44,45].

Figure 2b compares the experiments in which ultrasound time (U) and oxalic acid concentration (AO) were varied, while the UV-C distance (D) was fixed at 9 cm. The application of ultrasound alone (9D20U0AO) clearly reduced drying time. When only oxalic acid was applied (9D0U10AO), the process became even faster. The combination of both pretreatments (9D20U10AO) resulted in the greatest improvement, achieving the shortest drying time.

Figure 2c presents a similar comparison to Figure 2b, with the difference that D was set at 13 cm. In the condition 13D20U0AO, although ultrasound accelerated drying during most of the process, the time required to reach 15% moisture content (wet basis) was significantly higher. This finding highlights an antagonistic interaction between increasing both D and U simultaneously. Huang et al. [46] reported that ultrasound may also hinder drying depending on process conditions, food microstructure, and water uptake during treatment. In this case, the rapid initial moisture reduction may be associated with increased water content in the sample; however, the greater distance from the UV-C source likely reduced its effectiveness over time, leading to higher t_15_% values compared with other conditions. The remaining curves exhibited similar behavior, with the condition 13D0U10AO reaching the target moisture content the fastest, once again confirming the effectiveness of oxalic acid as a pretreatment. Figure 2d compares experiments in which ultrasound was not applied, while D and AO varied. As expected, conditions involving oxalic acid resulted in faster drying. A similar trend is observed in Figure 2e, where D and U varied while AO remained constant, showing that ultrasound application also enhanced drying rates.

To describe the drying behavior, empirical models were fitted to the experimental data [27]. Table 4 presents the parameters of the Henderson and Pabis (HP), Logarithmic (L), Wang and Singh (WS), and Simple Exponential (SE) models fitted to the experimental data, along with the statistical indicators R^2^, *RSS*, *RMSE*, and *RSD*. Among the evaluated models, the Logarithmic model provided the best fit to the data, with R^2^ values higher than 0.9988 and the lowest *RSS*, *RMSE*, and *RSD* values across all conditions. This indicates that the model accurately captures the falling-rate period characteristic of plantain peel drying, where moisture migration is primarily governed by internal diffusion. Similar findings have been reported in the literature, where the Logarithmic model showed superior performance in describing the drying behavior of fruit and vegetable residues, such as lemon peels [47], calabura [48], banana [7] and pomegranate seeds [49].

### 3.3. Effective Moisture Diffusivity

During drying, moisture migrates from the interior of the material to its surface, driven by a concentration gradient that may induce structural changes. The effective moisture diffusivity (*D_ef_*) quantifies this internal mass transfer rate [50].

The diffusivity analysis using the “Goal Seek” method was conducted up to a maximum of 200 min (12,000 s). As shown in Figure 2f, this time range was sufficient to evaluate the behavior of *D_ef_* throughout the drying kinetics. The generated curve clearly reveals three distinct stages. At the beginning of the process, diffusivity exhibits higher values, which decrease during the initial minutes until reaching a constant value (characteristic of the falling-rate period). After a certain time, closer to the end of drying, *D_ef_* values begin to increase again. Therefore, for all evaluated conditions, diffusivity values between 900 and 6000 s were considered for determining the average effective diffusivity (*D_efM_*), as presented in Table 5. The same interval was also used to estimate D_ef_ through mathematical modeling of the dimensionless moisture ratio data, also reported in Table 5.

The results in Table 5 confirm the observations reported by Silva et al. [21], emphasizing that effective diffusivity should be treated as constant only within a specific time interval to satisfy the assumptions of Fick’s model, namely, a phenomenological approach based on the falling-rate period, rather than assuming constant diffusivity over the entire drying process, which corresponds to a purely empirical approach. In this study, when both methods were compared using only data points within the falling-rate period, the results were much closer, with percentage differences between *D_efM_* and *D_ef_* ranging from 24% to 27%. Although still notable, these discrepancies are significantly lower than those reported by Silva et al. [21], who found that using all kinetic data in the empirical application of Fick’s equation led to D_ef_ values overestimated by more than two orders of magnitude.

It is also important to highlight that *D_efM_* values are more reliable, since *D_ef_* was obtained via mathematical modeling, with R^2^ values ranging from 0.94 to 0.98, indicating that not all experimental data were adequately fitted by the selected model. In the present work, *D_efM_* values ranged from 6.76 × 10^−10^ to 8.21 × 10^−10^ m^2^/s, which is consistent with values reported in the literature. For example, Han et al. [51] reported effective diffusivity values on the order of 10^−10^ m^2^/s during infrared-assisted convective drying of Chinese dwarf bananas.

Regarding the experimental trends, the application of ultrasound and oxalic acid pre-treatments significantly increased *D_efM_* compared to conditions in which samples were exposed only to UV-C radiation. This behavior is consistent with the previously discussed effects of ultrasound and oxalic acid on modifying the surface structure of plantain peels. A similar increase in effective diffusivity after pretreatment was also observed by Granella et al. [52], who studied ethanol- and ultrasound-treated dwarf banana samples.

### 3.4. Surface Morphological Analysis of Dried Peels

**Figure 3:**
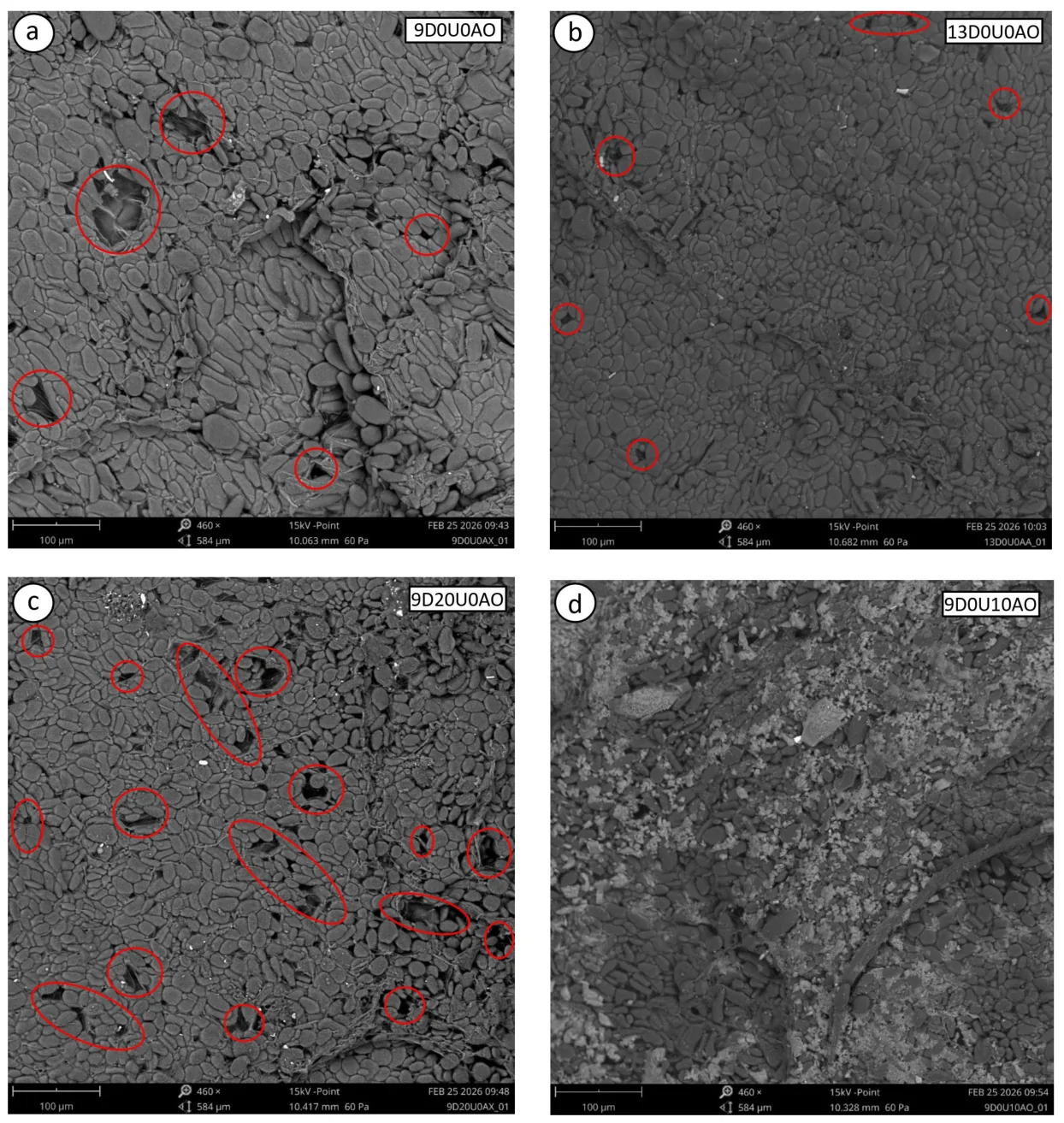
Scanning electron microscopy (SEM) micrographs. (**a**) Micrograph of condition 9D0U0AO. (**b**) Micrograph of condition 13D0U0AO. (**c**) Micrograph of condition 9D20U0AO. (**d**) Micrograph of condition 9D0U10AO. The red circles highlight regions affected by the applied treatments.

The analysis of images obtained via scanning electron microscopy (SEM) was carried out to identify the effects of UV-C radiation and pretreatments on the surface of dried plantain peels, and to understand how these factors may contribute to process intensification. Figure 3 presents micrographs of the conditions 9D0U0AO, 9D20U0AO, 9D0U10AO, and 13D0U0AO, which were selected to evaluate how variations in UV-C distance, along with the application of ultrasound and oxalic acid, influenced the surface structure of the samples. It should be emphasized that the microstructural assessment presented here is qualitative in nature: the micrographs provide visual evidence of morphological differences among conditions, but quantitative image analysis of pore size and pore density was not performed in this study. The observations below should therefore be interpreted with caution, and complementary quantitative analyses are recommended in future work.

By comparing Figure 3a,b, the effect of UV-C radiation during drying can be observed: in condition 9D0U0AO, larger and more widely distributed pores appear across the surface, whereas in 13D0U0AO the structure seems more compact, with fewer apparent microchannels, a condition that would be less favorable to moisture migration from the interior to the surface. These observations are consistent with the shorter drying times obtained at the shorter UV-C distance. Phonyiam et al. [53], who applied UV-C radiation to tangerine peels as a fungal control strategy, also reported the formation of pores in irradiated samples, with pore size and distribution influenced by radiation intensity. Nevertheless, SEM alone does not allow the conclusion that UV-C caused the formation of these pores, as other factors, such as the different drying times, may also have contributed to the observed morphology.

Figure 3c illustrates the effect of ultrasound on dried plantain peels. The structure of the ultrasound-treated sample appears less compact, with an apparently higher pore density compared to the other conditions. This observation is consistent with the significant effect of ultrasound in reducing drying time and with previous reports describing cavitation-induced structural changes during ultrasound application [13,54]. As in the case of UV-C, however, this evidence is qualitative, and the specific contribution of ultrasound to pore formation was not quantitatively confirmed.

Figure 3d corresponds to condition 9D0U10AO and shows the effect of oxalic acid on the sample structure. The impact of this treatment appears more pronounced than that of the other conditions, with an apparently more disrupted and porous surface. A plausible explanation is that the acid promotes hydrolysis of surface starch granules and disruption of cell wall components, which would facilitate moisture removal and accelerate drying; this hypothesis is consistent with the finding that oxalic acid concentration was the most significant factor in reducing *t_15%_* in the factorial design and with previous reports on the microstructural effects of organic acid pretreatments [55]. It should be noted, however, that scanning electron microscopy does not provide direct chemical evidence of starch hydrolysis, and this interpretation should therefore be regarded as a hypothesis to be confirmed by complementary analyses (e.g., starch staining or X-ray diffraction).

Overall, the qualitative microstructural observations are consistent with the drying kinetics and diffusivity results, suggesting that structural modifications of the plantain peel surface contributed to the enhanced moisture transport observed for the pretreated samples. Nevertheless, because quantitative image analysis of pore size and pore density was not performed, the specific contribution of each technology to pore formation and to moisture transport cannot be directly confirmed from the SEM data alone.

### 3.5. Quality Analysis

Quality analyses were performed only for six factorial conditions selected from the experimental design. The selection was based on two criteria: (i) the factorial conditions representing the main effects and interactions that were statistically significant for t_15%_ in the Pareto analysis, and (ii) the coverage of the factorial extremes of the experimental domain. Accordingly, experiments 9D0U0AO and 9D20U0AO were selected because the individual effects of UV-C distance (D) and ultrasound time (U), as well as their interaction, were found to be significant. The conditions 9D20U10AO and 13D20U10AO were included because the interaction among all three variables (D, U, and AO) was statistically significant. Experiments 9D0U10AO and 13D0U10AO were selected because, although the D×AO interaction was not significant, oxalic acid concentration (AO) showed the highest standardized effect in the Pareto chart. For comparison, analyses were also conducted on fresh unripe plantain peels and on a control sample dried by hot air at 60 °C without pretreatments and without UV-C radiation. It should be noted, however, that the selection was based on the significance pattern of t_15%_; since the bioactive, color, and water activity responses may follow a different significance pattern than the drying time, this selection limits the representativeness of the quality dataset.

#### 3.5.1. Water Activity (a_w_)

Water activity (a_w_) is a key parameter for assessing the quality of dried products. Since it is directly associated with the growth of spoilage and pathogenic microorganisms, its value must remain below 0.60 to ensure microbial inhibition [56]. Therefore, the water activity of both fresh and dried samples was determined, and the results are presented in Table 6.

The water activity of fresh unripe plantain peel was 0.98, as expected for an unprocessed product. Similar values have been reported in the literature for fresh materials, such as papaya seeds [57] and apple cubes [58], which also showed a_w_ values close to 0.98 prior to drying. In contrast, the flours produced from peels subjected to ultrasound and oxalic acid pretreatments, followed by UV-C-assisted drying, showed a substantial reduction in water activity, with values ranging from 0.44 to 0.59. These values fall within the expected range for dried products and indicate improved microbiological stability.

A similar trend was reported by Dhake et al. [59] in the drying of ripe banana peels (Nendran cultivar). In that study, samples were pretreated with potassium metabisulfite solutions (0.5% and 1.0%) and dried at different temperatures. At 60 °C, water activity values ranged from 0.28 to 0.34, confirming a significant reduction in a_w_ after drying.

#### 3.5.2. Color

Color is a highly sensitive quality attribute in fruits and vegetables, and it is strongly affected by drying methods. During processing, the most significant color degradation is associated with hot air application, which can lead to pigment breakdown due to polyphenol oxidation, as well as promote non-enzymatic browning reactions such as caramelization and the Maillard reaction [60,61]. The colorimetric results for unripe plantain peel flours are presented in Table 6.

**Figure 4:**
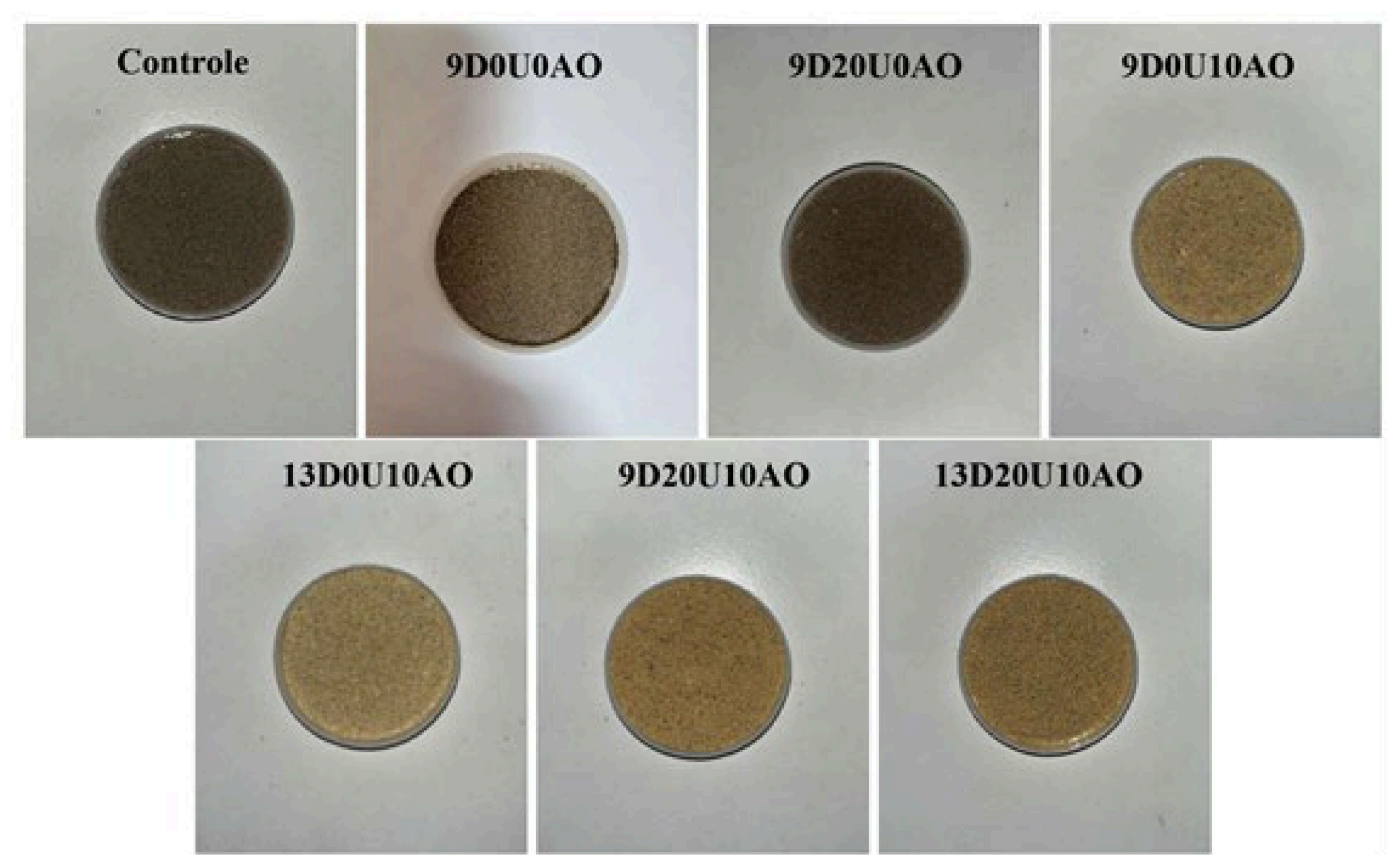
Plantain flour. The abbreviations D, U, and AO indicate the distance between the radiation source and the sample, the ultrasound application time, and the oxalic acid concentration, respectively.

From the analysis, it can be observed that UV-C radiation, either applied alone or combined with ultrasound, did not cause significant changes in lightness (L*) compared to the control sample. However, a marked increase in L* values was observed in samples pretreated with oxalic acid. Higher L* values indicate lighter-colored samples, which was also visually confirmed (Figure 4). This increase in lightness may be attributed to the inhibitory effect of oxalic acid on enzymes such as peroxidase (POD) and polyphenol oxidase (PPO), which are associated with enzymatic browning [62]. Two mechanisms have been proposed to explain this effect: (i) inhibition through chelation or removal of copper ions from PPO active sites, and (ii) pH reduction in the treated material, which decreases POD and PPO activity [63]. In addition, the impact of the Maillard reaction may have been reduced, since it is favored at higher pH values, whereas oxalic acid lowers the pH of the samples [64]. Similar anti-browning effects of oxalic acid have been reported by Arif et al. [65] in abiu fruit and by Huang et al. [66] in bananas, where treated samples showed higher L* values during storage, indicating reduced enzymatic browning.

A similar pattern was observed for the a* and b* parameters. No significant differences were found between the control and samples treated with UV-C radiation or ultrasound. However, samples pretreated with oxalic acid showed a significant increase in both parameters. Positive a* and b* values indicate a shift toward red and yellow hues, respectively, with higher values representing stronger color intensity [67].

Sun et al. [29], in a study on convective drying of potatoes pretreated with citric acid solutions, also reported increased a* values in treated samples compared to untreated ones, associating this effect with the occurrence of the Maillard reaction. Thus, it can be inferred that oxalic acid may slow down the Maillard reaction sufficiently to prevent overall darkening (i.e., reduction in L*), while not completely inhibiting it, resulting in a slight increase in reddish tones. On the other hand, Seerangurayar et al. [68] reported a decrease in b* values in dried dates, attributing this to the degradation of yellow pigments such as carotenoids, which leads to darker coloration. In contrast, the increase in b* observed in oxalic acid-treated samples in this study suggests that the treatment helps preserve pigments associated with yellow coloration, which also contributes to the higher L* values.

Regarding the total color difference (ΔE), since it is a function of L*, a*, and b*, only minor differences were observed between the control flour and those obtained under conditions 9D0U0AO and 9D20U0AO. In contrast, flours produced from oxalic acid-treated samples exhibited a pronounced color difference. As color variation directly influences consumer perception [69], this outcome is favorable in practical terms. As shown in Figure 4, lighter flours present a more appealing appearance compared to darker ones, enhancing their visual acceptability and potential for consumption.

#### 3.5.3. Total Phenolic Compounds, Ascorbic Acid, Total Carotenoids, and Antioxidant Capacity

Both fruit peels and pulps are widely recognized as rich sources of bioactive compounds such as polyphenols, carotenoids, and ascorbic acid. These substances are closely associated with antioxidant properties and can provide several health benefits by mitigating cellular oxidative stress [70,71]. However, drying processes often lead to substantial losses of these compounds. Therefore, quantifying the retained bioactive content after processing is essential for ensuring product quality and consumer acceptance [72]. The values of total phenolic content, ascorbic acid, total carotenoids, and antioxidant capacity of the plantain peel flours obtained under different conditions are presented in Table 7.

The total phenolic content of fresh unripe plantain peel was 45.76 mg GAE/g DM (equivalent to 4.67 mg GAE/g fresh sample), slightly higher than the value reported by Rodrigues et al. [1] for the Prata variety (1.45 mg GAE/g). This variation can be attributed to differences in cultivar, harvest conditions, climate, and soil, which influence fruit composition [27]. As expected, drying led to a reduction in total phenolics in most samples. The lowest values were observed in the control, 9D0U0AO, and 9D20U0AO conditions (4.70–9.43 mg GAE/g DM), with no statistically significant differences among them. These values are comparable to those reported by Viana et al. [73] for unripe banana peel flour dried at 55 °C.

In contrast, all samples pretreated with oxalic acid showed a marked increase in total phenolic content. The values for 9D0U10AO and 13D0U10AO were 22.52 and 26.27 mg GAE/g DM, respectively, with no significant difference between them, indicating that UV-C distance did not influence phenolic content under these conditions. The highest values were observed for 13D20U10AO and 9D20U10AO (37.49 and 51.36 mg GAE/g DM, respectively), highlighting the effect of combining all three factors. Notably, although the value obtained for 9D20U10AO exceeded that of the fresh sample, no statistically significant difference was observed.

The higher phenolic contents of the pretreated flours are most plausibly explained by the enhanced extractability of phenolic compounds after the structural disruption promoted by ultrasound and oxalic acid, which weakens the bonds between phenolics and the food matrix [74,75]. UV-C may additionally contribute to the retention of phenolic compounds by reducing their oxidative degradation during drying [27]. Although UV-C has been reported to trigger phenolic biosynthesis in living plant tissues [76,77], the samples in this study were actively dehydrating at 60 °C for 2.5–4.5 h, and no enzymatic or biosynthetic response was measured; therefore, active biosynthesis cannot be confirmed and was not assumed in this work.

Thus, the combined application of ultrasound, oxalic acid, and UV-C radiation favored the retention of phenolic compounds through complementary mechanisms: structural disruption (ultrasound and acid), which enhances extractability, and the shorter drying times, which reduce the thermal exposure of the samples. The superior performance of condition 9D20U10AO indicates that shorter UV-C distances and the most intense levels of ultrasound and oxalic acid minimized the degradation of phenolic compounds during drying.

The ascorbic acid content of fresh plantain peel was 35.76 mg/100 g DM. After drying, a significant reduction was observed in the control sample (7.40 mg/100 g DM), similar to the value reported by Egbuonu et al. [78]. No significant differences were found among the control, 9D0U0AO, and 9D20U0AO conditions. However, all samples treated with oxalic acid showed significantly higher ascorbic acid content (15.53–18.48 mg/100 g DM), indicating that oxalic acid was the main factor responsible for its preservation. This effect is likely due to its ability to stabilize vitamin C by forming complexes with metal ions that catalyze its degradation [79].

Carotenoids, another important class of bioactive compounds, are natural pigments responsible for yellow, orange, red, and purple colors in plant materials. They are essential for human health due to their provitamin A activity, antioxidant properties, and role in immune support [80,81]. The total carotenoid content of fresh samples was 71.47 µg/g DM (7.29 µg/g fresh basis), within the range reported by Aquino et al. [82].

After drying, most conditions showed a reduction in carotenoid content relative to the fresh peel. Among the samples pretreated with oxalic acid alone, the highest retention was observed for 13D0U10AO (63.13 µg/g DM) and the lowest content for 9D0U10AO (34.84 µg/g DM), suggesting that the shorter UV-C distance was associated with greater carotenoid degradation when ultrasound was not applied. Conversely, when ultrasound was combined with oxalic acid, the highest carotenoid content was found at the shortest distance (9D20U10AO, 77.94 µg/g DM), above the fresh level. These results indicate that the effect of UV-C distance on carotenoid retention depended on the combination of pretreatments applied.

The control and 9D0U0AO samples showed similar carotenoid levels (44.77 and 49.45 µg/g DM), indicating that UV-C alone did not significantly affect this parameter. However, the 9D20U0AO condition showed a notable increase (63.13 µg/g DM) compared with the control, suggesting that the combination of ultrasound and the shorter UV-C distance contributed to carotenoid preservation, possibly through the shorter drying time and the correspondingly reduced oxidative exposure [83,84]. Once again, the highest carotenoid content was observed in condition 9D20U10AO (77.94 µg/g DM), reinforcing the effect of combining all three variables. This result is consistent with the color parameter b*, which was also highest under this condition, reflecting the association between carotenoids and yellow pigmentation.

All the bioactive compounds discussed can contribute to antioxidant capacity by limiting the formation of reactive oxygen species (ROS). In human health, antioxidants help neutralize free radicals, reducing the risk of various diseases [85]. The antioxidant capacity of fresh plantain peel was 19.38 mg TE/g DM (1.98 mg TE/g fresh basis), consistent with values reported by Gallage et al. [86].

After drying, antioxidant capacity decreased significantly. The lowest value was observed in 9D20U0AO (2.66 mg TE/g DM), indicating that ultrasound negatively affected this parameter. This may be due to ROS generation during acoustic cavitation, which promotes oxidative degradation of bioactive compounds [87]. In contrast, oxalic acid pretreatment proved effective in preserving antioxidant capacity. All acid-treated samples showed similar values (5.54–5.81 mg TE/g DM), higher than the control (3.89 mg TE/g DM). This protective effect is likely due to the ability of oxalic acid to chelate metal ions that catalyze ROS formation, thereby reducing oxidative degradation of bioactive compounds [63].

To identify which of the analyzed bioactive compounds contributed significantly to the measured antioxidant capacity, a multiple linear regression analysis was performed. In this model, antioxidant capacity was considered the dependent variable, with the other compounds serving as independent variables. The fitted model was highly significant, with a p-value lower than 0.001. Thus, at least one of the bioactive compounds showed a linear relationship with antioxidant capacity. It is also worth noting that the model yielded an R^2^ value of 0.724, indicating that the levels of total phenolics, ascorbic acid, and carotenoids collectively explain 72.4% of the variability in antioxidant capacity.

However, an analysis of the individual contribution of each variable revealed that only the ascorbic acid content had a significant effect on antioxidant capacity, showing a partial correlation of 0.770 (*p* < 0.001). In contrast, the levels of total phenolic compounds and total carotenoids did not show a significant contribution to antioxidant capacity when adjusted within the multiple linear regression model, with p-values well above 0.05.

Therefore, the results of this study indicate that, under the conditions evaluated, ascorbic acid played a predominant role regarding antioxidant capacity. Since specific phenolic compounds and carotenoids were not individually determined, only their total amounts, it is reasonable to assume that the quantified compounds, as a group, exhibit low antioxidant capacity.

The high antioxidant capacity observed in condition 9D0U0AX, which showed a low ascorbic acid content, may be attributed to unquantified compounds that also possess antioxidant activity. In turn, the low antioxidant capacity observed in the 9D20U0AX condition, which exhibited a higher ascorbic acid content, may be related to the absence of a protective agent for ascorbic acid under this condition, as occurs in the samples pretreated with oxalic acid. Consequently, ascorbic acid may undergo rapid oxidation within the methanolic extract, thereby reducing its ability to scavenge DPPH during the analysis [63,79].

## 4. Conclusions

This study showed that the combination of UV-C-assisted hot-air drying with ultrasound and oxalic acid pretreatments intensified the drying of unripe plantain peels. The factorial design and drying kinetics analysis revealed that the UV-C distance, the ultrasound exposure time, and the oxalic acid concentration significantly affected the drying time, with shorter drying times being generally associated with shorter UV-C distances, longer ultrasound exposure, and higher oxalic acid concentrations.

The Logarithmic model provided the best description of the falling-rate period, and the effective moisture diffusivity, on the order of 10^−10^ m^2^/s, increased with the application of pretreatments. Scanning electron microscopy (SEM) provided qualitative evidence of structural modifications, including surface pores and microchannels, consistent with enhanced moisture transport.

All flours presented water activity below 0.60, which is compatible with microbiological stability; nevertheless, microbiological stability was not directly measured and should be confirmed in future studies. Pretreated flours, particularly those treated with oxalic acid, showed lighter color and higher contents of total phenolics, ascorbic acid, total carotenoids, and DPPH antioxidant capacity than the control, although the per-dry-matter values partially reflect the concentration of solids during drying.

The main limitations of this study are the screening nature of the factorial design, performed without replication of the corner points; the restriction of the quality analyses to a subset of the factorial conditions; the residual oxalate content of the pretreated flours, which was not measured in this study, although the samples were rinsed after the pretreatment, and should be quantified before the intended food application; the qualitative nature of the microstructural assessment; and the absence of sensory, storage, energy, and scale-up evaluations. In addition, the UV-C irradiance at the sample surface and the cumulative UV-C fluence were not measured, and the effect of UV-C distance was interpreted qualitatively; direct radiometric measurements are recommended in future studies. Future studies should therefore confirm the selected condition via response-surface optimization and experimental validation, evaluate strategies to reduce residual oxalate, and assess energy consumption, scale-up feasibility, storage stability, sensory acceptance, and the bioavailability of the bioactive compounds.

## Figures and Tables

**Table 1:** 2^3^ Factorial design matrix with drying conditions.

Experiment	D (cm)	U (min)	AO (% m/m)	Codification
1	9	0	0	9D0U0AO
2	13	0	0	13D0U0AO
3	9	20	0	9D20U0AO
4	13	20	0	13D20U0AO
5	9	0	10	9D0U10AO
6	13	0	10	13D0U10AO
7	9	20	10	9D20U10AO
8	13	20	10	13D20U10AO
9	11	10	5	11D10U5AO
10	11	10	5	11D10U5AO
11	11	10	5	11D10U5AO

The abbreviations D, U and AO indicate the distance between the radiation source and the sample, the ultrasound application time, and the oxalic acid concentration, respectively.

**Table 2:** Mathematical models for drying kinetics.

Model	Equation
Single exponential	MR=exp(−kt)
Henderson and Pabis	MR=a exp(−kt)
Logarithmic	MR=a exp(−kt)+c
Wang and Singh	$MR=1+at+bt^{2}$

*k*, *a*, *b*, *c*: parameters in thin-layer models; *MR*: moisture ratio.

**Table 3:** Sample drying times.

Sample	*t_15%_* (min)	Reduction Compared to 9D0U0AO (%)
9D0U0AO	263	-
9D20U0AO	193	26.62
9D0U10AO	201	23.57
9D20U10AO	156	40.68
13D0U0AO	235	10.65
13D20U0AO	233	11.41
13D0U10AO	162	38.40
13D20U10AO	206	21.67
11D10U5AO	195	25.86
11D10U5AO	195	25.48
11D10U5AO	196	25.86

The abbreviations D, U, and AO indicate the distance between the radiation source and the sample, the ultrasound application time, and the oxalic acid concentration, respectively.

**Table 4:** Parameters of the models fitted to unripe plantain peel drying data.

**Models**	**Samples**	**Parameters**				**R^2^**	***RSS***	***RMSE*** **× 10^2^**	***RSD***
Henderson and Pabis		***a***	***k***						
	9D0U0AO	0.9964	0.0158			0.9982	0.0314	0.0121	0.0110
	9D20U0AO	1.0053	0.0173			0.9969	0.0421	0.0219	0.0148
	9D0U10AO	1.0127	0.0184			0.9966	0.0492	0.0246	0.0157
	9D20U10AO	1.0065	0.0182			0.9946	0.0616	0.0390	0.0197
	13D0U0AO	1.0171	0.0170			0.9972	0.0474	0.0202	0.0142
	13D20U0AO	1.0114	0.0198			0.9988	0.0189	0.0081	0.0090
	13D0U10AO	0.9999	0.0166			0.9952	0.0544	0.0338	0.0184
	13D20U10AO	1.0069	0.0174			0.9971	0.0426	0.0208	0.0144
	11D10U5AO	0.9992	0.0164			0.9964	0.0499	0.0252	0.0159
	11D10U5AO	0.9883	0.0176			0.9972	0.0378	0.0194	0.0139
	11D10U5AO	0.9879	0.0189			0.9970	0.0385	0.0202	0.0142
Logarithmic		*a*	*k*	*c*		R^2^	*RSS*	*RMSE* × 10^2^	*RSD*
	9D0U0AO	1.0011	0.0149	−0.0150		0.9988	0.0214	0.0082	0.0090
	9D20U0AO	1.0268	0.0152	−0.0450		0.9993	0.0100	0.0053	0.0072
	9D0U10AO	1.0294	0.0163	−0.0393		0.9990	0.0137	0.0069	0.0083
	9D20U10AO	1.0580	0.0147	−0.0855		0.9992	0.0090	0.0057	0.0076
	13D0U0AO	1.0281	0.0154	−0.0305		0.9991	0.0156	0.0067	0.0082
	13D20U0AO	1.0162	0.0191	−0.0102		0.9991	0.0135	0.0059	0.0076
	13D0U10AO	1.0563	0.0134	−0.0882		0.9991	0.0102	0.0064	0.0080
	13D20U10AO	1.0246	0.0154	−0.0410		0.9995	0.0069	0.0034	0.0058
	11D10U5AO	1.0248	0.0142	−0.0512		0.9992	0.0110	0.0056	0.0075
	11D10U5AO	1.0048	0.0158	−0.0369		0.9990	0.0127	0.0066	0.0081
	11D10U5AO	1.0013	0.0170	−0.0329		0.9988	0.0161	0.0085	0.0092
Wang and Singh		*a*	*b* × 10^3^			R^2^	*RSS*	*RMSE* × 10^2^	*RSD*
	9D0U0AO	−0.0105	0.0272			0.9611	0.6788	0.2591	0.0509
	9D20U0AO	−0.0124	0.0395			0.9804	0.2676	0.1394	0.0374
	9D0U10AO	−0.0127	0.0405			0.9806	0.2786	0.1393	0.0373
	9D20U10AO	−0.0135	0.0481			0.9864	0.1543	0.0976	0.0312
	13D0U0AO	−0.0113	0.0319			0.9743	0.4280	0.1829	0.0428
	13D20U0AO	−0.0124	0.0373			0.9497	0.7803	0.3363	0.0580
	13D0U10AO	−0.0127	0.0431			0.9864	0.1528	0.0949	0.0308
	13D20U10AO	−0.0121	0.0374			0.9774	0.3293	0.1606	0.0401
	11D10U5AO	−0.0119	0.0364			0.9805	0.2725	0.1377	0.0371
	11D10U5AO	−0.0126	0.0409			0.9737	0.3518	0.1804	0.0425
	11D10U5AO	−0.0132	0.0444			0.9671	0.4285	0.2243	0.0474
Single exponential		*k*				R^2^	*RSS*	*RMSE* × 10^2^	*RSD*
	9D0U0AO	0.0158				0.9982	0.0381	0.0145	0.0120
	9D20U0AO	0.0172				0.9969	0.0425	0.0220	0.0148
	9D0U10AO	0.0181				0.9964	0.0515	0.0256	0.0160
	9D20U10AO	0.0181				0.9945	0.0623	0.0392	0.0198
	13D0U0AO	0.0167				0.9969	0.0519	0.0221	0.0149
	13D20U0AO	0.0195				0.9986	0.0214	0.0092	0.0096
	13D0U10AO	0.0166				0.9952	0.0544	0.0336	0.0183
	13D20U10AO	0.0173				0.9970	0.0433	0.0210	0.0145
	11D10U5AO	0.0164				0.9964	0.0500	0.0251	0.0158
	11D10U5AO	0.0179				0.9970	0.0398	0.0203	0.0143

The abbreviations D, U, and AO indicate the distance between the radiation source and the sample, the ultrasound application time, and the oxalic acid concentration, respectively.

**Table 5:** Comparison between the effective diffusivity estimated using the Levenberg–Marquardt method and the average effective diffusivity obtained using the “Goal Seek” method.

Sample	*D_ef_* × 10^10^ (m^2^/s)	R^2^	*D_efM_* × 10^10^ (m^2^/s)	Variation Between *D_efM_* and *D_ef_* (%)
9D0U0AO	5.05	0.9439	6.76	25.25
9D20U0AO	5.40	0.9592	7.24	25.39
9D0U10AO	5.71	0.9489	7.57	24.57
9D20U10AO	5.70	0.9555	7.57	24.72
13D0U0AO	5.17	0.9482	6.99	26.00
13D20U0AO	6.34	0.9533	8.21	22.79
13D0U10AO	5.15	0.9559	6.99	26.35
13D20U10AO	5.43	0.9543	7.27	25.30
11D10U5AO	5.08	0.9597	6.93	26.67
11D10U5AO	5.66	0.9669	7.59	25.45
11D10U5AO	6.15	0.9732	8.10	24.07

The abbreviations D, U, and AO indicate the distance between the radiation source and the sample, the ultrasound application time, and the oxalic acid concentration, respectively.

**Table 6:** Water activity and color analyses of fresh, control, and pretreated samples subjected to UV-C radiation during drying.

Samples	Water Activity (a_w_)	Color			
		*L*	a*	b*	∆*E*
Fresh	0.98 ± 0.02 ^a^	-	-	-	-
Control	0.45 ± 0.05 ^b^	37.45 ± 0.10 ^a^	3.52 ± 0.10 ^a^	10.12 ± 0.31 ^a^	-
9D0U0AO	0.44 ± 0.04 ^b^	36.08 ± 0.43 ^a^	3.64 ± 0.03 ^a^	11.27 ± 0.20 ^a^	1.86 ± 0.08 ^a^
9D20U0AO	0.44 ± 0.03 ^b^	37.81 ± 0.76 ^a^	3.41 ± 0.29 ^a^	10.53 ± 0.85 ^a^	1.07 ± 0.79 ^a^
9D0U10AO	0.48 ± 0.04 ^b^	64.22 ± 1.36 ^b^	4.62 ± 0.11 ^b,d^	17.88 ± 0.42 ^b^	27.90 ± 0.82 ^b^
9D20U10AO	0.59 ± 0.03 ^c^	65.60 ± 2.23 ^b^	5.52 ± 0.23 ^c^	23.28 ± 0.81 ^c^	31.14 ± 1.65 ^b,c^
13D0U10AO	0.51 ± 0.03 ^b,c^	70.57 ± 1.30 ^c^	4.34 ± 0.20 ^d^	21.07 ± 0.48 ^c^	34.90 ± 1.69 ^c^
13D20U10AO	0.45 ± 0.02 ^b^	64.54 ± 2.27 ^b^	5.15 ± 0.32 ^b,c^	22.38 ± 2.00 ^c^	29.79 ± 2.77 ^b^

The abbreviations D, U, and AO indicate the distance between the radiation source and the sample, the ultrasound application time, and the oxalic acid concentration, respectively. Mean *±* standard deviation (SD) with the same letter within the same column showed no statistically significant difference for their mean values at a 95% confidence level.

**Table 7:** Content of bioactive compounds in fresh, control, and pretreated samples subjected to UV-C radiation during drying.

Samples	Total Phenolics Content (mg GAE/g DM)	Ascorbic Acid Content (mg/100 g DM)	Total Carotenoids Content (µg/g DM)	Antioxidant Capacity (mg TE/g DM)
Fresh	45.76 ± 4.03 ^a^	35.76 ± 2.38 ^a^	71.47 ± 3.49 ^a^	19.38 ± 0.05 ^a^
Control	4.70 ± 0.48 ^b^	7.40 ± 0.92 ^b^	44.77 ± 1.08 ^b^	3.89 ± 0.12 ^b^
9D0U0AO	9.43 ± 1.52 ^b^	8.60 ± 0.48 ^b^	49.45 ± 0.74 ^b^	5.89 ± 0.10 ^c^
9D20U0AO	5.85 ± 0.48 ^b^	13.42 ± 1.16 ^b,c^	63.13 ± 2.06 ^c^	2.66 ± 0.01 ^d^
9D0U10AO	22.52 ± 2.73 ^c^	16.76 ± 2.23 ^c^	34.84 ± 1.75 ^d^	5.60 ± 0.02 ^e^
9D20U10AO	51.36 ± 2.01 ^a^	15.53 ± 3.36 ^c^	77.94 ± 2.23 ^e^	5.54 ± 0.01 ^e^
13D0U10AO	26.27 ± 2.95 ^c^	17.93 ± 3.29 ^c^	63.40 ± 1.40 ^c^	5.65 ± 0.01 ^e,f^
13D20U10AO	37.49 ± 1.19 ^d^	18.48 ± 1.62 ^c^	45.52 ± 2.44 ^b^	5.81 ± 0.06 ^c,f^

The abbreviations D, U, and AO indicate the distance between the radiation source and the sample, the ultrasound application time, and the oxalic acid concentration, respectively. Mean *±* standard deviation (SD) with the same letter within the same column showed no statistically significant difference for their mean values at a 95% confidence level.

## Data Availability

The original contributions presented in the study are included in the article. Further inquiries can be directed to the corresponding author.
